# Early Pregnancy Biomarkers in Pre-Eclampsia: A Systematic Review and Meta-Analysis

**DOI:** 10.3390/ijms160923035

**Published:** 2015-09-23

**Authors:** Pensée Wu, Caroline van den Berg, Zarko Alfirevic, Shaughn O’Brien, Maria Röthlisberger, Philip Newton Baker, Louise C. Kenny, Karolina Kublickiene, Johannes J. Duvekot

**Affiliations:** 1 Institute for Science and Technology in Medicine-Keele University, Guy Hilton Research Centre, Thornburrow Drive, Hartshill, Stoke-on-Trent ST4 7QB, UK; E-Mails: p.wu@keele.ac.uk (P.W.); shaughn.o’brien@uhns.nhs.uk (S.O.); 2 Academic Unit of Obstetrics and Gynaecology, Royal Stoke University Hospital, Maternity Centre, Newcastle Road, Hartshill, Stoke-on-Trent ST4 6QG, UK; 3 Department of Obstetrics and Gynecology, Subdivision of Obstetrics and Prenatal Medicine, Erasmus MC-University Medical Centre, PO Box 2040, 3000 CA Rotterdam, The Netherlands; E-Mail: c.vandenberg@erasmusmc.nl; 4 Department of Women’s and Children’s Health, The University of Liverpool, Liverpool L8 7SS, UK; E-Mail: zarko@liverpool.ac.uk; 5 Department of Obstetrics and Gynecology, University Hospital of Cologne, 50931 Cologne, Germany; E-Mail: maria.roethlisberger@uk-koeln.de; 6 College of Medicine, Biological Sciences and Psychology, University of Leicester, PO Box 138, Leicester LE1 9HN, UK; E-Mail: philip.baker@le.ac.uk; 7 Department of Obstetrics and Gynaecology, Cork University Maternity Hospital (5th Floor), Cork University Hospital, Wilton, Cork T12 YE02, Ireland; E-Mail: l.kenny@ucc.ie; 8 Karolinska Institutet, Centre for Gender Medicine, Institutions of Medicine and Clinical Science, Intervention and Technology, Department Ob/Gyn, Karolinska University Hospital, 14186 Stockholm, Sweden; E-Mail: karolina.kublickiene@ki.se

**Keywords:** pre-eclampsia, early pregnancy biomarkers, meta-analysis

## Abstract

Pre-eclampsia (PE) complicates 2%–8% of all pregnancies and is an important cause of perinatal morbidity and mortality worldwide. In order to reduce these complications and to develop possible treatment modalities, it is important to identify women at risk of developing PE. The use of biomarkers in early pregnancy would allow appropriate stratification into high and low risk pregnancies for the purpose of defining surveillance in pregnancy and to administer interventions. We used formal methods for a systematic review and meta-analyses to assess the accuracy of all biomarkers that have been evaluated so far during the first and early second trimester of pregnancy to predict PE. We found low predictive values using individual biomarkers which included a disintegrin and metalloprotease 12 (ADAM-12), inhibin-A, pregnancy associated plasma protein A (PAPP-A), placental growth factor (PlGF) and placental protein 13 (PP-13). The pooled sensitivity of all single biomarkers was 0.40 (95% CI 0.39–0.41) at a false positive rate of 10%. The area under the Summary of Receiver Operating Characteristics Curve (SROC) was 0.786 (SE 0.02). When a combination model was used, the predictive value improved to an area under the SROC of 0.893 (SE 0.03). In conclusion, although there are multiple potential biomarkers for PE their efficacy has been inconsistent and comparisons are difficult because of heterogeneity between different studies. Therefore, there is an urgent need for high quality, large-scale multicentre research in biomarkers for PE so that the best predictive marker(s) can be identified in order to improve the management of women destined to develop PE.

## 1. Introduction

Pre-eclampsia (PE) is an important cause of perinatal morbidity and mortality and complicates 2%–8% of pregnancies [1]. Worldwide, PE is responsible for more than 50,000 maternal deaths annually [2,3]. It is characterized by *de novo* hypertension and proteinuria after 20 weeks of gestation. However, PE continues to cause diagnostic dilemmas due to the heterogeneity of its clinical presentations. Clinical phenotypes range from early-onset severe hypertension accompanied by fetal growth restriction and its consequences to late-onset mild hypertension with a normally grown (or even macrosomic) fetus and few long-term complications. The concept that PE may involve several subtypes is now emerging in the literature. It is thought that the end clinical presentation may be due to the maternal response to abnormal placentation or placental function [4].

As PE cannot be predicted by previous obstetric history and risk factors alone [5], much research has focused on the identification of women at high risk of developing PE. This would allow more intensive monitoring of this high risk group as well as targeted prophylactic intervention, timely diagnosis and treatment. The identification of PE biomarkers in early pregnancy would enable appropriate stratification of a pregnancy into high and low risk, such that a positive predictive test would allow specific therapeutic interventions. Maternal deaths due to PE might thus be avoided more easily as the ultimate long term goal [6]. However, on a pragmatic basis, the identification of PE biomarkers would lead to increased maternal surveillance of high risk pregnancies and improve perinatal outcomes.

Due to the complex pathophysiology and aetiology of PE, a wide range of potential biomarkers have been investigated [7]. These biomarkers can be classified under different categories and many novel biomolecules have been identified. In addition to the predictive value of biomarkers, the identification of these entities (e.g., metabolomic or proteiomic molecules) may elucidate the underlying mechanism for the pathogenesis of PE. Although no single biomarker has been deemed suitable for clinical application at present [8] various novel biomarkers or combinations of biomarkers with other well recognized clinical parameters are promising. To this end, we conducted a systematic review and meta-analyses of biomarkers during the first half of pregnancy for the prediction of PE.

## 2. Results

**Figure 1:**
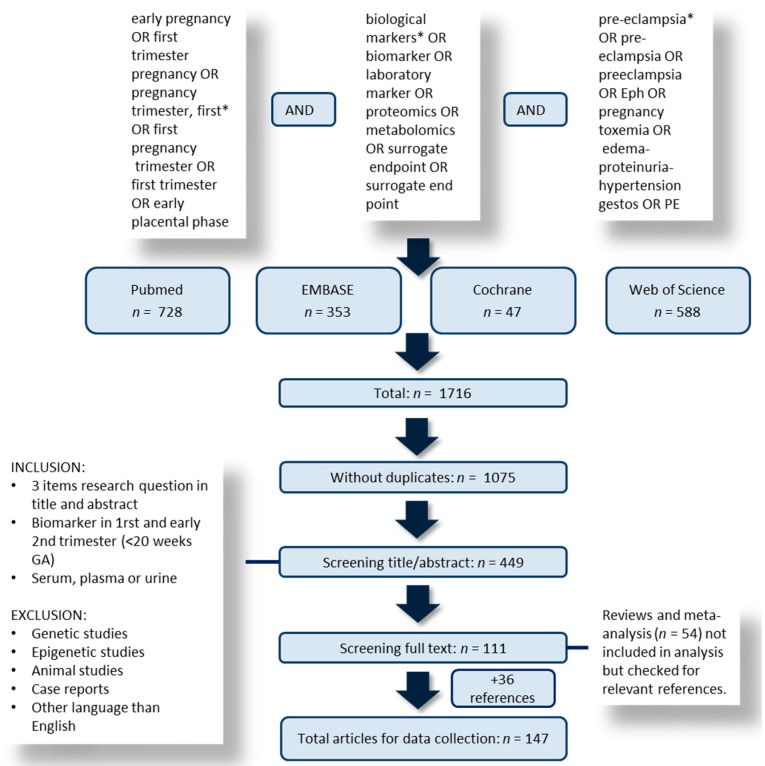
Flowchart of selection process. GA: gestational age.

**Figure 2:**
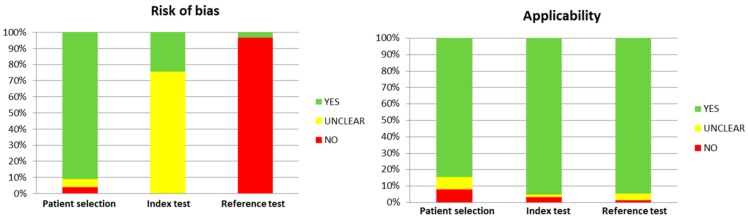
QUADAS-2 Quality score. QUADAS: Quality Assessment of Diagnostic Accuracy Studies.

**Figure 3:**
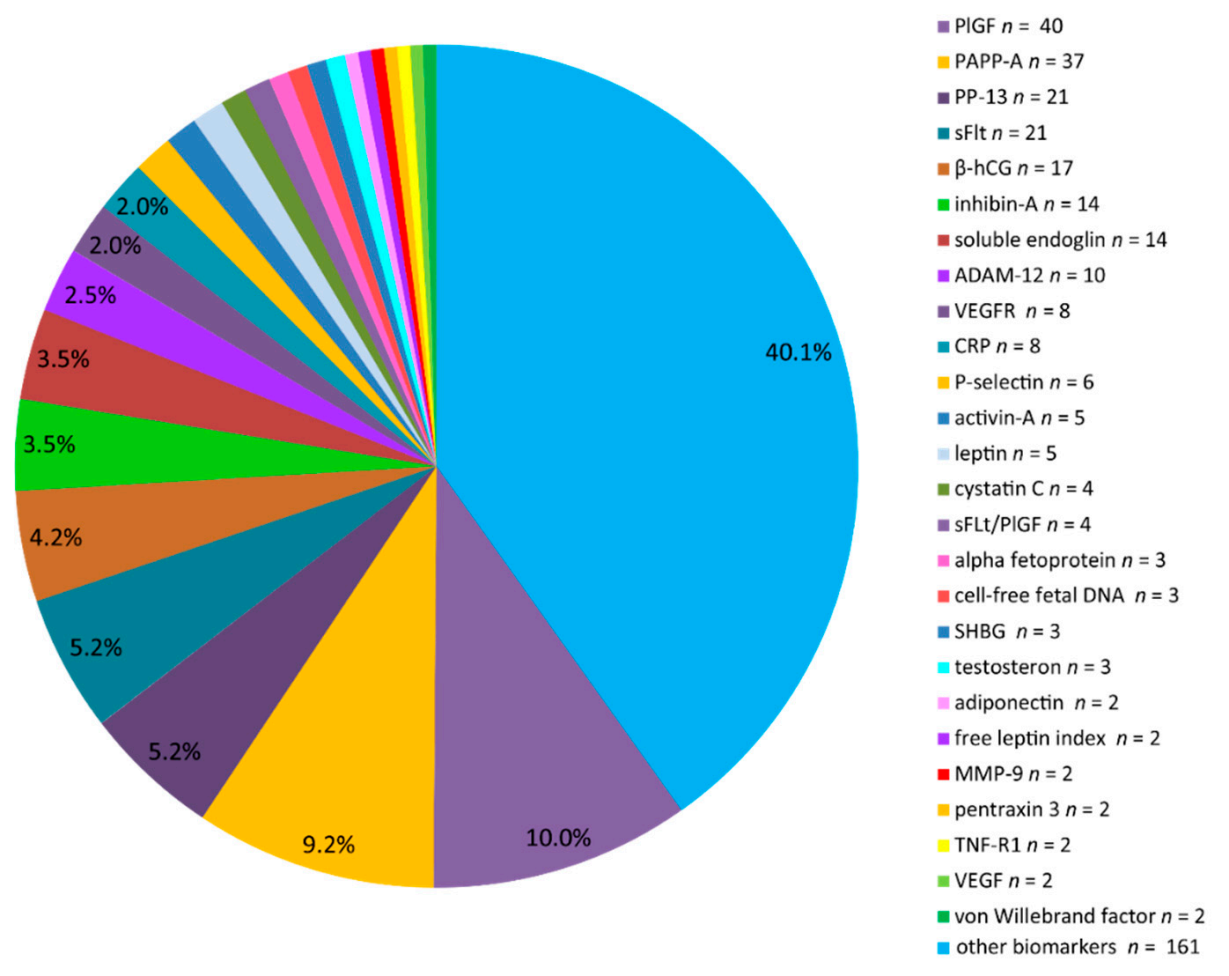
Distribution of studied laboratory biomarkers (*n* = 401) in included articles (*n* = 147). PlGF: Placental growth factor; PAPP-A: Pregnancy associated plasma protein A; PP-13: Placental protein 13; ADAM-12: a disintegrin and metalloprotease 12; CRP: C-reactive protein; sFlt: Soluble fms-like tyrosine kinase-1; MMP-9: Matrix metallopeptidase 9; TNF-R1: Tumour-necrosis factor receptor-1; VEGF: Vascular endothelial growth factor; VEGFR: Vascular endothelial growth factor receptor; SHBG: Sex hormone-binding globulin.

Of the 1716 identified articles, 147 articles were included following full screening. The study selection process is illustrated in Figure 1, while the overall result of the QUADAS-2 quality assessment is shown in Figure 2. Figure 3 demonstrates the frequency of the different laboratory biomarkers in all included studies (401 laboratory biomarkers were described in 147 studies). Placental growth factor (PlGF), pregnancy associated plasma protein A (PAPP-A), soluble fms-like tyrosine kinase (sFLT) and placental protein 13 (PP-13) were the most commonly studied biomarkers.

We were able to extract sensitivity and specificity from 36 studies for all PE, 10 studies for EOPE and 7 studies for LOPE; we performed a meta-analyses of all single biomarkers and of the reported combination of biomarkers separately. We performed separate meta-analyses for the following biomarkers (>2 studies available): a disintegrin and metalloprotease 12 (ADAM-12), inhibin-A, PAPP-A, PlGF, PP-13. The characteristics of the included studies are shown in Table 1.

**Table 1:** General characteristics of the included studies in the meta-analyses. GH: gestational hypertension; SGA: small for gestational age. PTB: preterm birth. The outcomes used were in line with the definitions from International Society for the study of Hypertension (ISSHP) [9].

Study	Year	GA of Test (Weeks)	Biomarker (s)	Outcome	Study Design ^A^	Low/High Risk	Location	*n* (Total)	*n* (PE)	Level of Evidence
Anderson *et al*. [10]	2011	11–16	α-1-microglobulin and fetal hemoglobin	PE	Nested case control (in prospective study) ^1^	LR	UK	96	60	3b
Akolekar *et al*. [11]	2008	11–14	PlGF, PAPP-A	EOPE, LOPE, GH	Nested case-control (in trisomy 21 screening cohort) ^2^	LR + HR	UK	824	127	3b
Akolekar *et al*. [12]	2013	11–14	PlGF, PAPP-A	PE	Prospective cohort (in screening)	LR + HR	UK	58,703	1245	1b
Audibert *et al*. [13]	2010	11–13	PAPP-A, ADAM-12, PlGF, hCG, inhibin-A, PP-13, protein-A, inhibin-A	PE, EOPE, LOPE, GH	Prospective cohort (trisomy 21 screening cohort) ^3^	LR + HR	Canada	893	40	1b
Bills *et al*. [14]	2009	First trimester	VEGF(165)b, sFLT, sEng	PE, EOPE, LOPE	Case-control ^4^	LR + HR	UK	70	25	3b
Bosio *et al*. [15]	2001	10–14	P-selectin	PE, GH	Nested case-control (in longitudinal cohort) ^5^	LR + HR	Ireland	70	20	3b
Boucoiran *et al*. (1) [16]	2013	12–18	PlGF, sFlt-1, inhibin A	PE, GH, SGA	Prospective cohort (nested in RCT) ^6^	LR + HR	Canada	793	34	1b
Boucoiran *et al.* (2) [17]	2013	11-14 and 18-22	PlGF, PP-13, ADAM-12	EOPE, LOPE, GH	Prospective cohort (trisomy 21 screening cohort) ^7^	LR + HR	Canada	893	40	1b
Brameld *et al*. [18]	2008	12 + 3	PAPP-A, free-hCG	PE	Retrospective cohort (trisomy 21 screening cohort) ^8^	LR	Australia	22,125	660	2b
Chafetz *et al*. [19]	2007	9–12	PP-13	PE, PTB, SGA	Nested case control in prospective cohort (MOMS-study) ^9^	LR	USA	425	47	3b
Cohen *et al*. [20]	2014	10–13	PAPP-A, α fetoprotein, free β-hCG	PE	Nested case control (retrospective cohort) ^10^	LR + HR	USA	2199	148	3b
Cowans *et al.* [21]	2011	11–14	PP-13	EOPE, LOPE	Nested case control (in cohort of trisomy screening) ^11^	HR	UK	234	37	3b
Deurloo *et al*. [22]	2013	9–14	ADAM-12, PP-13	PE, GH, SGA	Nested case control (in cohort of trisomy screening ^12^	LR + HR	The Netherlands	220	17	3b
Dugoff *et al.* [23]	2004	10–14	PAPP-A	PE, PTB, SGA	Prospective study (FASTER trial, trisomy screening cohort) ^13^	LR	USA	34,271	764	1b
Giguere *et al*. [24]	2014	10–18	PlGF, sFlt, PAPP-A, inhibin-A	PE	Nested case-control (in prospective cohort) ^14^	LR	Canada	648	216	3b
Goetzinger *et al*. [25]	2013	11–14	ADAM-12, PAPP-A	PE, EOPE, LOPE	Prospective cohort ^15^	LR + HR	USA	578	54	1b
Gonen *et al.* [26]	2008	6–10	PP-13	PE, GH	Prospective cohort ^16^	LR + HR	Israel	1239	20	1b
Ghosh *et al*. [27]	2013	11–14	PlGF	EOPE	Prospective study (screening antenatal care) ^17^	LR + HR	India	1206	9	1b
Hedley *et al*. [28]	2010	10–14	PAPP-A, free leptin index	PE	Nested case control (in First Trimester Screening Study) ^18^	LR	Denmark	415	126	3b
Kang *et al*. [29]	2008	11 and 16	PAPP-A, AFP, uE3, hCG, inhibin-A	PE	Retrospective cohort (trisomy 21 screening cohort) ^19^	LR + HR	Korea	3076	32	2b
Kenny *et al*. [30]	2014	14–16	Multiple	PE, EOPE, preterm and term PE	Prospective cohort ^20^	LR	Australia/UK/Ireland	5623	278	1b
Khalil *et al*. [31]	2010	11–14	PP-13	PE, EOPE, PE + SGA	Nested case-control (in antenatal clinic cohort) ^21^	HR	UK	252	42	3b
Kuc *et al.* [32]	2013	9–14	PAPP-A, free -hCG, ADAM-12, PlGF	EOPE, LOPE	Nested case control (in screening cohort) ^22^	LR + HR	The Netherlands	667	167	3b
Kusanovic *et al.* [33]	2009	6–15	PlGF, soluble endoglin, sVEGFR-1	EOPE, LOPE	Prospective cohort ^23^	LR	Chile	1622	62	3b
Myatt *et al*. [34]	2012	9–13	ADAM-12, PAPP-A, PP-13, sFLT, endoglin	PE	Nested case control (in cohort of RCT) ^24^	LR	USA	683	174	2b
Myers *et al*. [35]	2013	14–16	PlGF, soluble endoglin, sFLT-1	preterm PE (<37 week)	Prospective cohort ^25^	LR	Australia/UK/Ireland	235	47	1b
Nicolaides *et al.* [36]	2006	11–14	PP-13	EOPE	Nested case control (in screening cohort) ^26^	LR + HR	UK	433	10	3b
Odibo *et al.* [37]	2011	11–14	PP13, PAPP-A	PE, EOPE	Prospective cohort (trisomy 21 screening cohort) ^27^	LR + HR	USA	452	42	1b
Park *et al*. [38]	2014	11–14	PAPP-A, PlGF, inhibin-A, sFLT	LOPE	Prospective cohort ^28^	LR	Korea	262	8	1b
Poon *et al*. (1) [39]	2009	11–14	PAPP-A	PE, EOPE, LOPE	Prospective cohort (trisomy 21 screening cohort) ^29^	LR + HR	UK	8051	156	1b
Poon *et al*. (2) [40]	2009	11–14	PAPP-A, MMP-9, TNF-R1	EOPE, LOPE, GH, SGA, PTB	Nested case-control (in trisomy 21 screening cohort) ^30^	LR + HR	UK	1138	128	3b
Roes *et al*. [41]	2004	6–15	Inhibin-A	PE	Case control ^31^	LR	The Netherlands	55	19	3b
Schneuer *et al*. [42]	2012	11–13	PP-13	PE, EOPE, LOPE, SGA	Prospective cohort (trisomy 21 screening cohort) ^32^	LR + HR	Australia	2678	71	1b
Spencer *et al*. [43]	2006	11–14	PP-13, PAPP-A	PE, EOPE, LOPE	Nested case-control (in trisomy 21 screening cohort) ^33^	LR	UK	534	88	3b
Spencer *et al.* [44]	2008	11–14	Inhibin-A and activin-A	PE, EOPE, LOPE	Nested case-control (in trisomy 21 screening cohort) ^34^	LR	UK	304	64	3b
Tidwell *et al*. [45]	2001	5–15	PlGF	EOPE, LOPE	Case control ^35^	LR	Taiwan	39	14	3b
Thilaganathan *et al*. [46]	2010	14.7 (CO), 16.3 (PE)	cystatin-C, CRP	PE	Nested case-control (in antenatal clinic cohort) ^36^	LR	UK	170	45	3b
Xu *et al.* [47]	2014	First trimester	Chemerin	PE	Prospective cohort (antenatal care)^37^	LR	China	518	41	1b
Youssef *et al.* [48]	2011	11–14	PAPP-A, PlGF, sFlt-1, P-selectin, NGAL	LOPE	Prospective cohort ^38^	LR + HR	Italy	528	13	1b
Yu *et al.* [49]	2011	12–16	PlGF, inhibin-A, activin-A	PE	Nested case-control (in antenatal clinic cohort) ^39^	LR	China	124	31	3b
Zong *et al.* [50]	2012	13–16	Htr-A1 (High-Temperature Requirement A1)	PE	Prospective cohort (clinical cohort) ^40^	LR	China	1396	100	1b

^A^ Characteristics of the study population are mentioned below; ^1^ Exclusion criteria diabetes, prepregnancy hypertension and premature delivery; ^2^ Controls: did not develop any pregnancy complications and resulted in the live birth of phenotypically normal neonates; ^3^ Exclusion: multiparous, multiple gestation, major fetal chromosomal/structural anomaly; ^4^ Exclusion: pregnancy induced hypertension, fetal growth restriction, intrauterine death, preterm birth (PTB); ^5^ Controls: normal obstetric outcome. Matched for body mass index (BMI); ^6^ Exclusion: vitamin C and/or vitamin E supplements, history of major medical complications, major fetal defects, repeated spontaneous abortion, use of an illicit drug or warfarin treatment during the current pregnancy; ^7^ Inclusion: nulliparous women with singleton pregnancies without major fetal chromosomal or structural anomaly; ^8^ Exclusion: Women who had a previous fetus with a chromosomal abnormality and women with insulin-dependent diabetes mellitus; ^9^ Exclusion: AIDS or hepatitis, cases of major fetal anomaly, fetal death and women with placenta previa, placenta accrete, or placental abruption; ^10^ General population, singleton pregnancies; ^11^ A priori high risk pregnancies; ^12^ General population; ^13^ Inclusion: singleton pregnancy, exclusion: diabetes and chromosomal abnormalities; ^14^ Exclusion: chronic hepatic or renal diseases, pregnancies with major fetal abnormalities and those ending in termination, miscarriage or fetal death <24 weeks; ^15^ Exclusion : known aneuploidy and major congenital malformations; ^16^ Exclusion: miscarriages; ^17^ Exclusion: congenital abnormalities or medication use; ^18^ Randomly selected controls; ^19^ Exclusion: multifetal gestation, diabetes, chromosomal or structural abnormalities; ^20^ Exclusion: increased risk factors of PE, SGA or PTB, known major fetal anomaly or abnormal karyotype, intervention that may modify pregnancy outcome such as treatment with aspirin or progesterone; ^21^ Inclusion: history of PE in a previous pregnancy, chronic hypertension, chronic renal disease, antiphospholipid syndrome, systemic lupus erythematosus, pregestational diabetes mellitus, obesity (BMI ≥ 30 kg/m^2^). Exclusion: multiple pregnancy, cases of major fetal anomaly, miscarriage or fetal death, HIV or hepatitis, placenta previa or placental abruption; ^22^ Exclusion: multiple pregnancy, delivery <24 weeks, chromosomal abnormalities; ^23^ Inclusion: pregnancies in which a single live fetus was delivered after 37 complete weeks of gestation with birth weight above the 10th centile and without fetal anomalies; ^24^ Inclusion: nulliparous, low risk women; ^25^ Exclusion: increased risk of PE, SGA or PTB, known major fetal anomaly or abnormal karyotype, intervention that may modify pregnancy outcome such as treatment with aspirin or progesterone; ^26^ Gestational age matched controls; ^27^ Inclusion : singleton pregnancies. Exclusion: spontaneous miscarriage prior to 20 weeks, loss to follow-up or fetal anomalies diagnosed in the second trimester; ^28^ Exclusion: high risk pregnancies; ^29^ Definition controls: randomly selected women without reported pregnancy-associated hypertension; ^30^ Definition controls: had blood collected and stored on the same day, which did not develop any pregnancy complications and resulted in the live birth of phenotypically normal neonates; ^31^ Unknown in- and exclusion criteria; ^32^ Inclusion: singleton pregnancies; ^33,34^ Gestational age matched controls; ^35^ Exclusion: multiparity, chronic hypertension, diabetes, multiple gestation, connective tissue disorder, any long-term use of medicine other than prenatal vitamins, and miscarriage before viability; ^36^ Exclusion: diabetes, connective tissue disease, renal disorders, essential hypertension; ^37^ Exclusion: previous systemic disorders or drug use, chronic hypertension, diabetes, renal disorders, recent or present fever or infectious disease, malignancies, autoimmune diseases and multiple pregnancies; ^38^ Exclusion: early-onset PE, multiple gestations, pregnancies with fetal chromosomal or major structural anomaly, miscarriages; ^39^ Unknown inclusion/exclusion criteria; ^40^ Exclusion: cases showing intrahepatic cholestasis of pregnancy, abortion, peripartum cardiomyopathy, and other complications. PlGF: Placental growth factor; PAPP-A: Pregnancy associated plasma protein A; ADAM-12: A disintegrin and metalloprotease 12; CRP: C-reactive protein; hCG: human chorionic gonadotropin; PP-13: Placental protein 13; VEGF: Vascular endothelial growth factor; sFLT: Soluble fms-like tyrosine kinase-1; sEng: Soluble endoglin; sVEGR-1: Soluble endothelial growth factor-1; uE3: Oestradiol; MMP-9: Matrix metallopeptidase 9; TNF-R1: Tumor-necrosis factor receptor-1; NGAL: Neutrophil gelatinase-associated lipocalin; (1): publication 1 by same author in same year; (2): publication 2 by same author in same year.

### 2.1. PE

**Figure 4:**
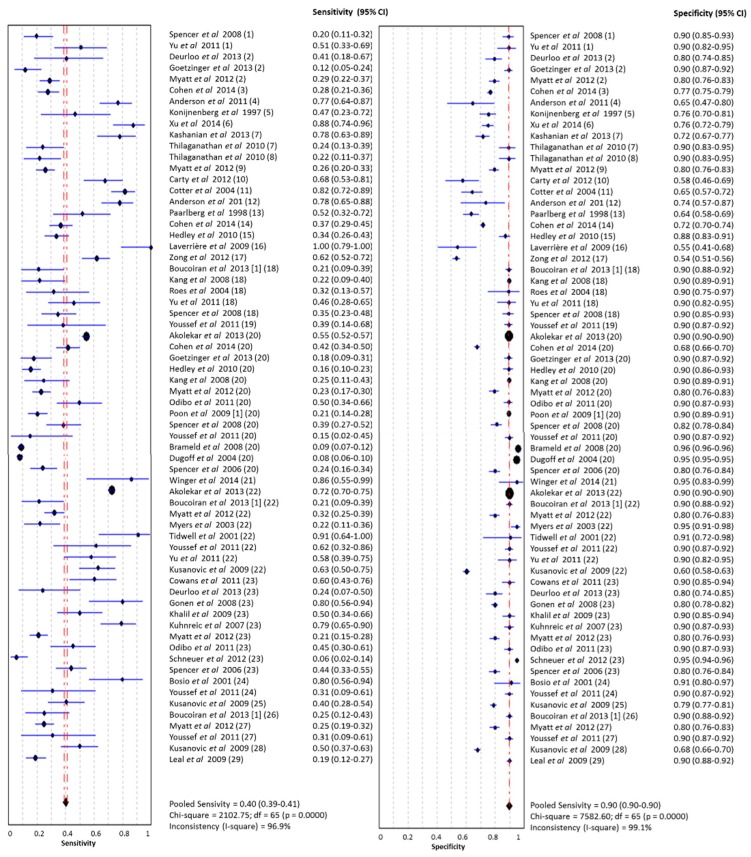
Meta-analysis of single laboratory biomarkers in PE (both EOPE and LOPE). Legend: (**1**) activin-A; (**2**) ADAM-12; (**3**) α fetoprotein; (**4**) α-1-macroglobulin; (**5**) anti-CD63 (GP53, lysosomal secretion); (**6**) chemerin; (**7**) C-reactive protein; (**8**) cystatin C; (**9**) endoglin; (**10**) E-selectin; (**11**) fetal DNA; (**12**) fetal hemoglobin (ratio); (**13**) fibronectin; (**14**) free β-hCG; (**15**) free leptin index; (**16**) GRP78 (glucose regulated protein) ratio C-term/full length; (**17**) Htr-A1 (High-Temperature Requirement A1); (**18**) inhibin-A; (**19**) NGAL (neutrophil gelatinase-associated lipocalin); (**20**) PAPP-A; (**21**) PBMC (peripheral blood mononuclear cell) miRNA; (**22**) PlGF; (**23**) PP-13; (**24**) P-selectin; (**25**) soluble endoglin; (**26**) sFLT/PlGF ratio; (**27**) sFlt-1; (**28**) sVEGFR-1 (vascular endothelial growth factor); (**29**) TNF-R1 (tumor necrosis factor receptor). PE: Pre-eclampsia; EOPE: early-onset PE; LOPE: late-onset PE.

**Figure 5:**
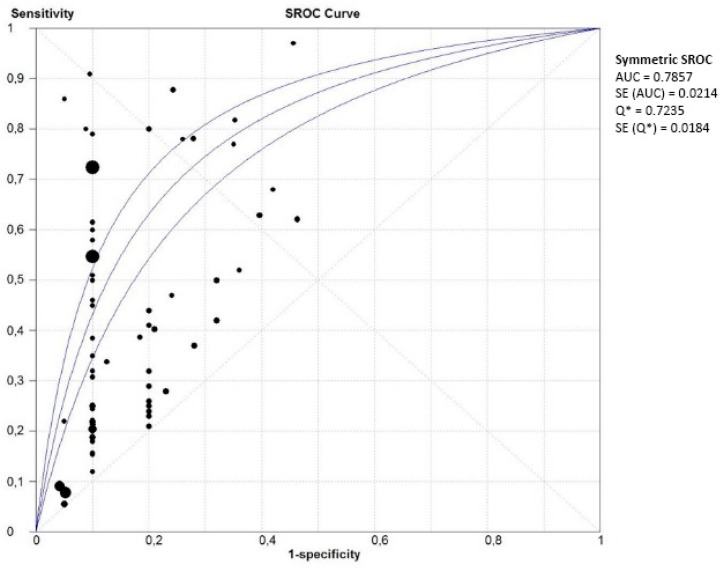
Summary of receiver operating characteristics curve of single laboratory biomarkers in PE (both EOPE and LOPE).

In studies which analysed women with PE without sub-classifying into EOPE and LOPE, the pooled sensitivity of all single biomarkers (*n* = 66) was 0.40 (95% CI 0.39–0.41, I^2^ 96.9%) at a false positive rate of 10% (Figure 4). The area under the SROC was 0.786 (SE 0.02) (Figure 5). The pooled sensitivity, specificity and area under the SROC of the separate meta-analyses of ADAM-12, inhibin-A, PAPP-A, PlGF and PP-13 are shown in Table 2. All these meta-analyses showed a high heterogeneity (I^2^ > 50%).

### 2.2. Early-Onset PE

In the group of studies which categorized EOPE separately (*n* = 17), the pooled sensitivity of all single biomarkers was 0.37 (95% CI 0.32–0.41, I^2^ 82.4%) with a specificity of 0.88 (95% CI 0.87–088, I^2^ 98.8%). The area under the SROC was 0.794 (SE 0.05). The pooled sensitivity, specificity and area under the SROC of the separate meta-analyses of PAPP-A, PlGF and PP-13 are shown in Table 2.

### 2.3. Late-Onset PE

In late-onset PE, (*n* = 14), the pooled sensitivity of all single biomarkers was 0.22 (95% CI 0.19–0.25, I^2^ 82.2%) with a specificity of 0.89 (95% CI 0.88–089, I^2^ 97.4%). The area under the SROC was 0.763 (SE 0.106). The pooled sensitivity, specificity and area under the SROC of the separate meta-analyses of PAPP-A is shown in Table 2.

### 2.4. Combination of Biomarkers

**Figure 6:**
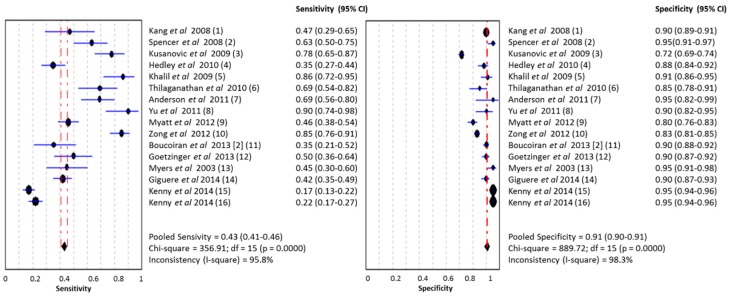
Meta-analysis of combination of laboratory and clinical makers in PE (both EOPE and LOPE). Legend: (**1**) PAPP-A, AFP, uE3, hCG (total or free β), inhibin-A; (**2**) mean PI + activin-A; (**3**) PlGF/sEng-ratio; (**4**) PAPP-A and free leptin index; (**5**) PP-13, UA-PI, AIx-75 (measure of arterial stiffness); (**6**) cystatin-C, CRP, uterine artery resistance index; (**7**) HbF ratio and A1M; (**8**) activin-A, inhibin-A, PlGF and UA-PI; (**9**) African American race, systolic blood pressure, BMI, education level, ADAM12, PAPP-A, PlGF; (**10**) BMI, education mother and HtrA1; (**11**) maternal characteristics, PlGF; (**12**) maternal characteristics, ADAM12; (**13**) maternal characteristics, PlGF; (**14**) sFLT-1, PlGF, PAPP-A, inhibin A, BMI, MAP; (**15**) PlGF, MAP, BMI, high fruit intake, uterine artery Doppler resistive index (UA-RI) * validation cohort; (**16**) PlGF, MAP, BMI, high fruit intake, UA-RI * training cohort.

**Figure 7:**
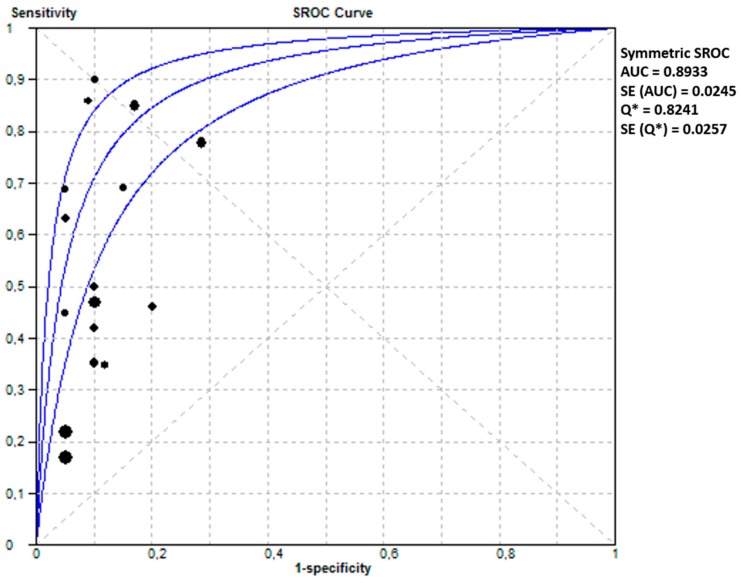
Summary of receiver operating characteristics curve of combination model of laboratory and makers in PE (both EOPE and LOPE).

From 13 studies, we extracted a pooled sensitivity of 0.43 (95% CI 0.41–0.46, I^2^ 95.8) and a pooled sensitivity of 0.91 (95% CI 0.90–0.91, I^2^ 98.3) and an area under the SROC of 0.893 (SE 0.03) for a combination model of clinical characteristics, laboratory biomarkers and/or uterine artery Doppler pulsatility index (UA-PI). The forest plot and SROC of these studies are shown in Figure 6 and Figure 7, respectively.

**Table 2:** Meta-analyses of single laboratory biomarkers.

All PE	Pooled Sensitivity (95% CI)	Pooled Specificity (95% CI)	Area Under SROC (SE)	EOPE	Pooled Sensitivity (95% CI)	Pooled Specificity (95% CI)	Area Under SROC (SE)	LOPE	Pooled Sensitivity (95% CI)	Pooled Specificity (95% CI)	Area Under SROC (SE)
ADAM-12 (*n* = 3)	0.26 (021–0.32)	0.84 (0.82–0.86)	0.671 (0.093)	ADAM-12 (*n* = 3)	-	-	-	-	-	-	-
Inhibin-A (*n* = 5)	0.32 (0.25–0.39)	0.90 (0.89–0.91)	0.957 (0.046)	Inhibin-A (*n* = 5)	-	-	-	-	-	-	-
PAPP-A (*n* = 14)	0.30 (0.29–0.32)	0.92 (0.92–0.92)	0.744 (0.071)	PAPP-A (*n* = 4)	0.26 (0.19–0.34)	0.90 (0.89–0.90)	0.907 (0.150)	PAPP-A (*n* = 4)	0.19 (0.14–0.24)	0.89 (0.89–0.90)	0.781 (0.173)
PlGF (*n* = 8)	0.65 (0.63–0.67)	0.89 (0.89–0.89)	0.849 (0.068)	PlGF (*n* = 3)	0.37 (0.27–0.48)	0.79 (0.78–0.81)	0.796 (0.179)	-	-	-	-
PP-13 (*n* = 9)	0.37 (0.33–0.41)	0.88 (0.87-0.89)	0.882 (0.0450)	PP-13 (*n* = 9)	0.59 (0.48–0.69)	0.92 (0.91–0.93)	0.898 (0.064)	-	-	-	-

## 3. Discussion

There is extensive literature on biomarkers in relation to PE and despite our focused strategy, we identified 401 biomarkers from the included publications. We then conducted a systematic review and meta-analyses using studies where we were able to extract comparable data for AUC and with more than two studies for each biomarker.

We examined single biomarkers in research conducted with different study cohorts, *i.e*., EOPE, LOPE or PE in general. Five biomarkers were highlighted: ADAM-12, inhibin-A, PAPP-A, PlGF and PP-13. ADAM12 is part of the ADAM protein family, which are involved in cell-to-cell and cell-to-matrix interactions in neural and muscle development as well as fertilization [51,52,53]. PAPP-A is part of the first trimester Down’s syndrome screening test and is a large zinc glycoprotein produced by placental trophoblasts [54]. PlGF and sFLT are both angiogenic factors. PlGF is a polypeptide growth factor mainly expressed in placental trophoblasts and regulate the early development of placental villi [55] while sFLT induces endothelial cell dysfunction [56].

Prediction models utilizing a combination of biomarkers and clinical parameters improved the predictive value in studies examining PE (without distinction of EOPE and LOPE) with an area under the SROC of 0.893. However, the majority of combined models include evaluation of clinical history or assessment of uterine artery Doppler waveforms. This limits the potential of solely using laboratory-based biomarkers.

A limitation of this study is that our search strategy lead to significant number of missed articles that were found subsequently by other means, such as through the reference lists of articles that have been already identified. This may be due to our limited search terms and the wide variation in terminology used for studies on PE. Previous meta-analyses on early pregnancy biomarkers for PE have concentrated on either biochemical markers alone [42] or in combination with ultrasound indices [57,58,59].

Due to the low population prevalence of PE, despite >200 studies on candidate biomarkers in the literature, none (nor any combination) have been identified with specificity and sensitivity that are useful for clinical practice [60]. The systematic review from the World Health Organization (WHO) concluded that there is no cost effective or reliable screening test (clinical, biophysical, or biochemical) for PE [61]. Perhaps this finding reflects that different types of biomarkers could point to different preventative strategies. For example, pregnancies associated with raised ADAM levels may need to be treated with aspirin, while those linked to raised PlGF levels may need to be treated with calcium.

The low predictive values using a single biomarker may be due to the heterogeneity between most studies such that we were unable to extract comparable data. Despite using the International Society for the study of Hypertension (ISSHP) definition in our review, there is a wide variation in the clinical manifestations and categorization of PE, such as early or late gestation, maternal or placental disease and mild or severe degree of PE. These could have introduced additional variability between the studies. Furthermore, many studies were conducted using different biomarkers, study population and definition of PE phenotype, *i.e.*, EOPE, LOPE or PE as one entity. We identified 147 articles but only 36 of these could be included in the meta-analyses. For each biomarker analyzed, the number of studies was even lower.

Many publications used PE, without sub-classification into EOPE and LOPE. This resulted in a poorly defined phenotype of PE which may further contribute to the low predictive value in these studies. As EOPE and LOPE have distinct and different pathogenesis mechanisms, it is likely that they are characterized by different biomarkers. Therefore, it is important to stratify study populations appropriately for accurate identification of biomarkers.

A possible source of bias arises from the over-representation of case-control studies in the reviewed literature. Furthermore, some studies were only conducted in women at high-risk of developing PE. Biomarkers which only have a high predictive value in EOPE may be another cause of overestimation. On the other hand, it is difficult to conduct studies focusing on LOPE as the phenotype is generally less severe than EOPE.

A well-designed study for biomarkers to predict PE in early pregnancy should be conducted in clearly defined populations, such as those with EOPE. The classic WHO screening criteria by Wilson and Jungner [62] can be adapted for biomarker studies [63,64]. These include: clearly defined clinical population and setting for use, set inclusion and/or exclusion criteria, focused outcome of interest, prospective specimen collection, aim for positive biomarker results in case and negative biomarker results in control, random selection of case and control subjects, accurate definition of true positive and true negative rates, clinically acceptable minimal test performance, favourable comparison with current risk stratification strategy, defined procedures for sample collection, processing, storage and retrieval, blind sampling, consideration of null hypothesis and alternative hypotheses, adequate sample size and that there is a policy present for early termination of the study if appropriate.Identification of women at risk of PE pre-eclampsia is the first step to effective intervention and prevention. However, currently there are no reliable biomarker tests for PE that have been accepted for wide clinical use and some countries have banned the use of biomarker screening in early gestation due to the possibility of inaccurate predictive test and its ethical implications. It is vital to develop a screening tool which is clinically relevant due to the serious consequences of incorrect risk stratification and inappropriate medication or pregnancy surveillance.

## 4. Methods

### 4.1. Studies

We searched MEDLINE, EMBASE, Cochrane and ISI web of science from inception to 31st January 2015 using a combination of search terms with synonyms related to pre-eclampsia (“preeclampsia”, “pre-eclampsia”, “EPH”, “pregnancy toxemia”, “edema-proteinura-hypertension gestos”, “PE”), biomarkers (“biological markers”, “biomarker”, “biological markers”, “laboratory markers”, “proteomics”, “metabolomics”, “surrogate endpoint”, “surrogate end point”), and first trimester pregnancy (“early pregnancy”, “first trimester pregnancy”, “pregnancy trimester, first”, “first pregnancy trimester”, “first trimester”, “early placental phase”). There were no limitations made on publication date or patient sample size. We excluded publications which were not in English. Animal studies were not included.

### 4.2. Study Selection

Two independent reviewers (Pensée Wu and Caroline van den Berg) screened the title, abstract and key words of each article and made a record of the study design, biomarker type, and test period during pregnancy and study outcome. We included observational studies (cohort, cross-sectional and case-control) which assessed tests performed in the first or early second trimester of pregnancy for predicting pre-eclampsia in unselected women. The outcome definitions were as described in the definition of PE from International Society for the study of Hypertension in Pregnancy (ISSHP).[9] Comments, editorials, case series (as defined by the authors of the studies) or reports were excluded, as were biomarker tests performed after 20 weeks of gestation. Genetic markers were not included as they require a different methodological approach and meta-analytic techniques. Reviews were included in the original search, to check for additional references.

### 4.3. Quality Assessment

An adapted version of the Quality Assessment of Diagnostic Accuracy Studies (QUADAS-2) tool was used to determine the methodological quality of the selected studies as not all items of the tools were relevant to our review [65,66]. Summary scores were not calculated as their interpretation is difficult and may be potentially misleading [67].

### 4.4. Data Extraction and Synthesis

We extracted the study outcome measures which were shown in the articles (odds ratio, risk ratio, area under the curve (AUC), sensitivity and specificity). In multiple or duplicate publication of the same data set, we used the most complete or the most recent study. To perform the meta-analyses we only used studies where a sensitivity and specificity was reported. We performed these meta-analyses separately for the three different outcomes: PE, early-onset PE (EOPE, before 34 weeks of gestation) and late-onset PE (LOPE, after 34 weeks of gestation). Studies that used biomarkers in combination with clinical parameters were analyzed separately.

Meta-DiSc (version 1.4; Zamora *et al.*, Madrid, Spain) [68] was used for statistical analyses. A pooled sensitivity and specificity was calculated, as well as a Summary of Receiver Operating Characteristics Curve (SROC). Raw data were used from each study, as adjustments for confounding effects varied between different studies. The inverse variance of the study was used to determine the weighting of studies in the meta-analyses. The random effects model was chosen due to the expected clinical and statistical heterogeneity among the studies. We assessed the heterogeneity of the results among studies through visual examination of Forest plots of AUC’s, and using the I^2^ test [69]. For all effect estimates, a value of *p <* 0.05 was considered to be statistically significant.

## 5. Conclusions

We found that PlGF was best at predicting EOPE as a single biomarker. However, a combination model performed better than a single biomarker if studying PE as a single entity. A combination model including clinical and uterine artery Doppler assessments, negates the attraction of using a laboratory-based biomarker(s) prediction strategy.

Despite multiple potential biomarkers for PE, the efficacy of these markers has been inconsistent between different studies. The IMPROvED (IMproved PRegnancy Outcomes by Early Detection) study is an international multicentre study screening 5000 women in five European countries with the aim of developing a clinically robust predictive blood test for PE, utilising novel metabolite and protein biomarkers [60]. We hope our study will contribute towards the ultimate goal of identifying the best predictive marker(s) and improve the management of women destined to develop PE.
